# Assessment of Employee Susceptibility to Phishing Attacks at US Health Care Institutions

**DOI:** 10.1001/jamanetworkopen.2019.0393

**Published:** 2019-03-08

**Authors:** William J. Gordon, Adam Wright, Ranjit Aiyagari, Leslie Corbo, Robert J. Glynn, Jigar Kadakia, Jack Kufahl, Christina Mazzone, James Noga, Mark Parkulo, Brad Sanford, Paul Scheib, Adam B. Landman

**Affiliations:** 1 Department of Medicine, Massachusetts General Hospital, Boston; 2 Division of General Internal Medicine and Primary Care, Brigham and Women’s Hospital, Boston, Massachusetts; 3 Partners HealthCare, Boston, Massachusetts; 4 Harvard Medical School, Boston, Massachusetts; 5 Division of Pediatric Cardiology, Department of Pediatrics & Communicable Diseases, University of Michigan Medical School, Ann Arbor; 6 Department of Cybersecurity, Utica College, Utica, New York; 7 Division of Preventive Medicine, Brigham and Women’s Hospital, Boston, Massachusetts; 8 Division of Information Assurance, University of Michigan Medical School, Ann Arbor; 9 Center for Translational Informatics and Knowledge Management, Mayo Clinic, Jacksonville, Florida; 10 Libraries and Information Technology Services: Enterprise Security, Emory University, Atlanta, Georgia; 11 Information Services Division, Boston Children’s Hospital, Boston, Massachusetts; 12 Department of Emergency Medicine, Brigham and Women’s Hospital, Boston, Massachusetts

## Abstract

**Importance:**

Cybersecurity is an increasingly important threat to health care delivery, and email phishing is a major attack vector against hospital employees.

**Objective:**

To describe the practice of phishing simulation and the extent to which health care employees are vulnerable to phishing simulations.

**Design, Setting, and Participants:**

Retrospective, multicenter quality improvement study of a convenience sample of 6 geographically dispersed US health care institutions that ran phishing simulations from August 1, 2011, through April 10, 2018. The specific institutions are anonymized herein for security and privacy concerns.

**Exposures:**

Simulated phishing emails received by employees at US health care institutions.

**Main Outcomes and Measures:**

Date of phishing campaign, campaign number, number of emails sent, number of emails clicked, and email content. Emails were classified into 3 categories (office related, personal, or information technology related).

**Results:**

The final study sample included 6 anonymized US health care institutions, 95 simulated phishing campaigns, and 2 971 945 emails, 422 062 of which were clicked (14.2%). The median institutional click rates for campaigns ranged from 7.4% (interquartile range [IQR], 5.8%-9.6%) to 30.7% (IQR, 25.2%-34.4%), with an overall median click rate of 16.7% (IQR, 8.3%-24.2%) across all campaigns and institutions. In the regression model, repeated phishing campaigns were associated with decreased odds of clicking on a subsequent phishing email (adjusted OR, 0.511; 95% CI, 0.382-0.685 for 6-10 campaigns; adjusted OR, 0.335; 95% CI, 0.282-0.398 for >10 campaigns).

**Conclusions and Relevance:**

Among a sample of US health care institutions that sent phishing simulations, almost 1 in 7 simulated emails sent were clicked on by employees. Increasing campaigns were associated with decreased odds of clicking on a phishing email, suggesting a potential benefit of phishing simulation and awareness. With cyberattacks increasing against US health care systems, these click rates represent a major cybersecurity risk for hospitals.

## Key Points

**Question:**

Are employees at US health care institutions susceptible to phishing attacks?

**Findings:**

In this multicenter quality improvement study, more than 2.9 million simulated emails were sent to employees at 6 hospitals, with a median click rate of 16.7%. Repeated phishing campaigns were associated with decreased odds of clicking on a subsequent phishing email.

**Meaning:**

Employees at US health care institutions may be susceptible to phishing emails, which presents a major cybersecurity risk to hospitals.

## Teaser

This multicenter quality improvement study type describes the practice of phishing simulation and the extent to which health care employees are vulnerable to phishing simulations.

## Introduction

The security of health care data and systems is rapidly emerging as a critical component of hospital infrastructure, and attacks on hospital information systems have had substantial consequences, with closed practices, canceled surgical procedures, diverted ambulances, disrupted operations, and damaged reputations.^1,2,3^ Attacks against hospitals have been increasing, with substantial financial cost as well.^4,5^ In a recent well-publicized example, a large hospital network was taken offline by a virus for almost 2 weeks, resulting in service disruption, patient confusion, and delays in radiation therapy, among other repercussions.^6^ Health care delivery has become increasingly dependent on integrated, complex information systems that are susceptible to disruption. Securing our health information systems is critical to safe and effective care delivery and is now of public health concern.^7^

Phishing is the practice of deceiving individuals into disclosing sensitive personal information or clicking on links that introduce malicious software through deceptive electronic communication.^8^ Usually done via email, phishing is a common attack strategy against health care system employees and can be a remarkably accessible, low-cost, and effective way of obtaining real credentials to health care information systems or inducing employees to click on malicious software.^9^ Phishing emails can be realistic, and the sender’s identity is frequently spoofed, or deliberately faked, so as to appear to be sent by a trusted individual or organization. Once an attacker has access to a system, they can steal personally identifiable information and sell it for profit, disrupt system availability, encrypt a database and demand a ransom payment to unlock it (“ransomware”), manipulate and falsify clinical data, or perform other malicious activities.^7^ A recent report indicated that 55% of physicians have experienced a phishing attack.^10^

Employee awareness and training represent an important component of protection against phishing attacks.^5^ One method of generating awareness and providing training is to send simulated phishing emails to a group of employees and subsequently target educational material to those who inappropriately click or enter their credentials. For reference, 2 examples of phishing emails are listed in eTable 1 in the Supplement. The first email is a phishing simulation, and the second is an actual phishing email received at 1 of the participating institutions. As shown, the emails can be realistic and often appear to be sent by a trusted individual or member of the employee’s organization. Phishing simulation is common in many industries and is also being used in health care, typically as a training and improvement initiative. The simulated emails are designed to be as close as possible to real phishing emails; if the simulated email is clicked, it is used as a real-time opportunity to provide short phishing education to the employee. Several vendors exist that offer phishing simulation as a service (eg, composing and sending the simulation emails, collecting employee responses, providing phishing training, and reporting on click rates to hospital leadership). In this context, we examined the practice of phishing simulation and the extent to which health care employees are vulnerable to phishing simulations and identified potential determinants of vulnerability to email phishing simulation.

## Methods

### Participants

In this retrospective, multicenter quality improvement study, we partnered with a sample of 6 US health care institutions that run phishing simulations using vendor- or custom-developed software tools. These institutions represent a diverse set of organizations across the entire spectrum of care and a range of US geographies, including institutions from the 4 US Census Bureau census regions; all had implemented an information security program. The identities of the specific institutions are anonymized herein for security and privacy concerns. Some participants were health care systems that operated multiple hospitals; in this case, we defined an institution as including multiple hospitals. More information about the institutions is listed in eTable 2 and eTable 3 in the Supplement. The Partners Healthcare Institutional Review Board determined the study to be exempt from review. The requirement of written informed consent was waived for the study. This study adhered to the Standards for Quality Improvement Reporting Excellence (SQUIRE) 2.0 reporting guideline.

### Data Collection

Data collected from participating institutions included institution, content of the phishing email, the number of emails delivered, and the number of clicks. Collaborators provided their data per phishing campaign, where a campaign was defined as an email with specific content sent to a group of employees. While individual employee characteristics were not available and responses of the same employee were not linked over time, no employees were excluded from phishing campaigns. All employees across all types of hospital roles (clinical and nonclinical) were eligible to receive the emails. One institution (site 2) ran several campaigns against small, targeted subsets of the population (eg, information security professionals). Because these campaigns were not general employee campaigns, they were excluded to increase generalizability.

### Email Classification

Because different phishing emails might be more likely to be clicked based on their content, we classified all emails into 1 of the following 3 categories: office related, personal, or information technology (IT) related. These categories were generated by consensus among 3 of us (W.J.G., A.W., and A.B.L.). Emails were then separately classified by 2 of us (W.J.G. and A.W.), and disagreements were refereed by another of us (A.B.L.). Examples of each email category are listed in Table 1.

**Table 1.** Email Category and Click Rates Among 95 Simulated Phishing Campaigns^a^

Email Category	Example Lures	No. (% Total) of Campaigns
Office related	You have received a new fax…	37 (38.9)
	You are expected to review this document on an annual basis…	
	Mandatory online workplace safety training…	
Personal	Someone sent you a Halloween e-card…	22 (23.2)
	Your new credit card has been shipped…	
	We are pleased to announce that you are eligible to receive double rewards…	
IT related	Your mailbox has exceeded the storage limit, which is 20 GB as set by your administrator…	36 (37.9)
	We are currently updating our database and email center. All unused accounts will be deleted…	
	If you are receiving this message, it means that your email address has been queued for deactivation…	

Abbreviation: IT, information technology.

^a^ Emails were placed into 1 of 3 categories based on expert review. Shown are example lures from each of the categories, highlighting the type of content that is used to solicit further engagement with the phishing email from employees. Also shown are the number of campaigns from our sample that fell into each category.

### Statistical Analysis

Institutions were anonymized (site 1 through site 6). The subsequent data set contained no institution- or person-identifiable information. We performed descriptive statistics on the institutions and phishing campaigns. We aggregated our data by institution and by campaign and calculated the proportion of emails that were clicked by employees, as well as the median click rates for each campaign. Multivariable logistic regression, with the use of a generalized estimating equation approach,^11^ was used to compute odds ratios (ORs) with 95% CIs for the odds that a phishing email would be clicked during a campaign. We used a generalized estimating equation approach with independence working correlation to obtain robust variance estimates because campaign click rates within an institution may be correlated. Covariates included year (2011-2018, centered on 0), the number of campaigns the institution had run before the phishing email being sent (institutional campaign number 1-5, 6-10, or >10), an indicator for anonymized institution, email category (office related, personal, or IT related), and season. All analyses were conducted using a software program (R; R Foundation for Statistical Computing).

## Results

**Figure 1.**
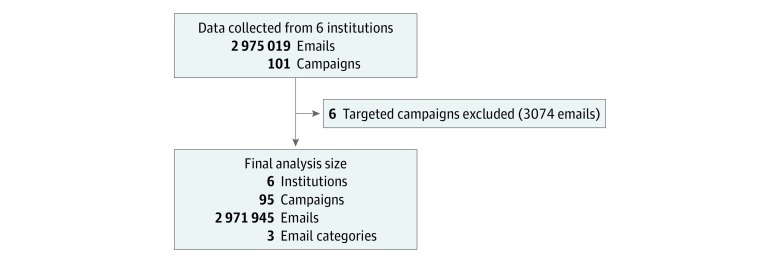
Study Design and Data Acquisition Data collected for each campaign included year of campaign, institutional campaign number, emails sent, emails clicked, and email category.

A convenience sample of 6 US health care institutions provided data for the study. These hospitals ran 101 simulated phishing campaigns and sent 2 975 019 emails from August 1, 2011, through April 10, 2018. After excluding 6 targeted campaigns (3074 emails), our final sample size included 95 campaigns and 2 971 945 emails (Figure 1). We classified the remaining emails into 1 of 3 categories (37 office related, 22 personal, and 36 IT related). Interrater reliability for categorization was high (Cohen κ = 0.746). The median click rates varied by email category, from 12.2% (interquartile range [IQR], 7.2%-20.7%) for office related to 18.6% (IQR, 13.9%-25.6%) for IT related (Table 2).

**Table 2.** Unadjusted Click Rates Among 95 Simulated Phishing Campaigns Across All Sites

Variable	No. of Campaigns	Unadjusted Click Rate, Median (IQR), %
Reference	95	16.7 (8.3-24.2)
Year (centered on 0)		
2011	12	23.5 (18.7-26.4)
2012	4	22.0 (15.2-30.8)
2013	None	NA
2014	None	NA
2015	18	19.1 (10.1-19.4)
2016	21	18.4 (10.3-25.0)
2017	33	9.9 (4.8-17.2)
2018	7	10.2 (7.3-15.7)
Institutional campaign No.		
1-5	28	25.1 (13.8-31.1)
6-10	19	17.9 (10.4-22.2)
>10	48	13.4 (6.3-18.8)
Institution		
Site 1 (n = 19)	19	10.2 (5.3-18.3)
Site 2 (n = 33)	33	14.5 (8.0-22.6)
Site 3 (n = 3)	3	7.4 (5.8-9.6)
Site 4 (n = 9)	9	14.5 (8.3-21.0)
Site 5 (n = 26)	26	19.0 (15.6-25.6)
Site 6 (n = 5)	5	30.7 (25.2-34.4)
Email category		
Office related	37	12.2 (7.2-20.7)
Personal	22	15.9 (6.8-24.5)
IT related	36	18.6 (13.9-25.6)
Season		
Fall	24	18.5 (13.4-22.2)
Winter	30	16.4 (9.7-21.9)
Spring	18	14.0 (8.1-25.5)
Summer	23	11.8 (5.7-28.1)

Abbreviations: IQR, interquartile range; IT, information technology; NA, not applicable.

**Figure 2.**
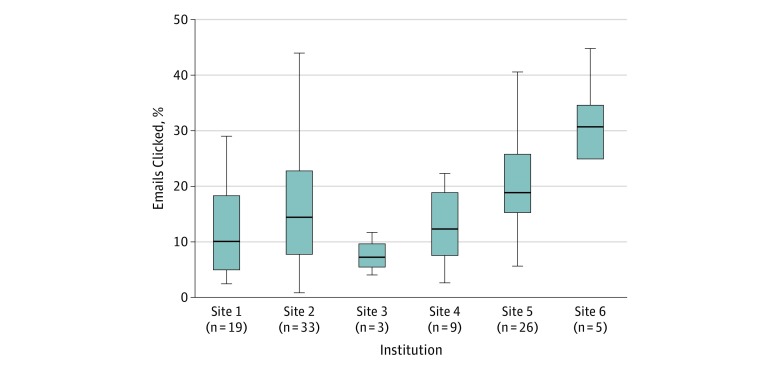
Boxplot of Campaign Click Rate Among 95 Simulated Phishing Campaigns, by Site The click rate distribution is shown by site. Each site is an anonymized institution. Click rate is calculated as a proportion (total emails sent divided by total emails delivered) across each campaign. The whiskers indicate the minimum and maximum values for each institution. The lower and upper borders of the box represent the first and third quartiles, respectively, while the line in the box represents the median.

The overall click rate across all institutions and campaigns was 14.2% (422 062 clicks per 2 971 945 emails). The median institutional click rates for campaigns ranged from 7.4% (IQR, 5.8%-9.6%) for site 3 to 30.7% (IQR, 25.2%-34.4%) for site 6, with an overall median click rate of 16.7% (IQR, 8.3%-24.2%) across all campaigns and institutions (Table 2 and Figure 2).

We found that repeated phishing campaigns were associated with decreased odds of clicking on a subsequent phishing email (adjusted OR, 0.511; 95% CI, 0.382-0.685 for 6-10 campaigns; adjusted OR, 0.335; 95% CI, 0.282-0.398 for >10 campaigns). Year did not have a significant association with click rates (adjusted OR, 0.965; 95% CI, 0.841-01.107). Click rates varied by institution, from an adjusted OR of 0.302 (95% CI, 0.225-0.406) compared with reference to an adjusted OR of 1.463 (95% CI, 1.299-1.648). Emails that were office related were not significantly associated with click rates (adjusted OR, 1.354; 95% CI, 0.865-2.120) compared with IT related (reference), but personal emails were significantly associated with increased click rates (adjusted OR, 1.505; 95% CI, 1.128-2.007) compared with IT related. Finally, certain seasons were associated with click rates. For example, both spring (adjusted OR, 0.842; 95% CI, 0.735-0.964) and summer (adjusted OR, 0.751; 95% CI, 0.624-0.905) campaigns were associated with fewer clicks compared with fall campaigns, while winter was not significantly associated with click rates (adjusted OR, 1.175; 95% CI, 0.972-1.420). Full results are listed in Table 3.

**Table 3.** Logistic Regression Model (With the Use of Generalized Estimating Equations) for the Odds of Clicking on 95 Simulated Phishing Campaigns

Variable	Model OR (95% CI)	*P* Value
Reference	0.279 (0.197-0.396)	<.001
Year (centered on 0)	0.965 (0.841-1.107)	.61
Institutional campaign No.		
1-5	1 [Reference]	NA
6-10	0.511 (0.382-0.685)	<.001
>10	0.335 (0.282-0.398)	<.001
Institution		
Site 1 (n = 19)	0.788 (0.651-0.954)	.02
Site 2 (n = 33)	1 [Reference]	NA
Site 3 (n = 3)	0.302 (0.225-0.406)	<.001
Site 4 (n = 9)	0.557 (0.474-0.654)	<.001
Site 5 (n = 26)	0.584 (0.393-0.868)	.008
Site 6 (n = 5)	1.463 (1.299-1.648)	<.001
Email category		
Office related	1.354 (0.865-2.120)	.19
Personal	1.505 (1.128-2.007)	.005
IT related	1 [Reference]	NA
Season		
Fall	1 [Reference]	NA
Winter	1.175 (0.972-1.420)	.10
Spring	0.842 (0.735-0.964)	.01
Summer	0.751 (0.624-0.905)	.003

Abbreviations: IT, information technology; NA, not applicable; OR, odds ratio.

## Discussion

In this study of US health care institutions that run phishing simulations, overall click rates varied by institution but were notably high: on average, almost 1 in 7 simulated emails sent were clicked on by employees. In models adjusted for several potential confounders, including year, institutional campaign number, institution, and email category, the odds of clicking on a phishing email were 0.511 lower for 6 to 10 campaigns at an institution and 0.335 lower for more than 10 campaigns at an institution. We also found that there were important institutional differences in click rates, as well as differences in click rates between email category and season.

Our study demonstrates that, similar to other industries,^12^ health care institutions conduct phishing simulations to raise awareness and identify employees who may benefit from education and training. We show herein that, under simulation, a large number of employees click on phishing emails, consistent with findings across other industries, where click rates can range from 13% to 49%, depending on industry.^13^ We found that the odds of clicking on a phishing email decreased with greater institutional experience, which we hypothesize may be due to the benefit of running phishing simulation campaigns for employee education and awareness. In addition, we note that there is a wide range of click rates between simulated campaigns. We hypothesize that the range of click rates is due to a number of factors, including prior employee exposure to phishing simulations (eg, from previous employment), complexities of individual phishing emails, email timing, and institutional factors (eg, messaging), as well as individual, employee-level factors that we were unable to collect or control for, which will need further study.

Health care systems have been increasingly targeted by cyberattacks, either as part of larger international events (eg, WannaCry or NotPetya)^1,7,14^ or as direct targets themselves.^2^ Health care delivery organizations are critical infrastructure and are attractive targets for cybercriminals for several reasons, including the value of personal health data (ranging from $10 to $1000 per record in online marketplaces, depending on completeness^15,16^), the criticality of services provided by hospitals,^17^ and an overall lack of information security processes.^18^ Phishing is an easily deployable attack strategy, largely because email is an easy access point to hospital employees, many of whom have credentials for several internal information systems (eg, electronic health records). In our experience, email addresses are easy to ascertain, either from published resources (journal articles, public websites, and social media) or through guessing (eg, firstname_lastname[at]hospital[dot]org). In addition, emails are frequently opened, regardless of sender. For example, more than one-third of sales and marketing emails are ultimately opened.^19^ The open rate may be even higher for emails that are not sales related.

Health care systems are also uniquely vulnerable to phishing attacks. Employee turnover at hospitals is high,^20^ and there is a constant influx of new employees (eg, trainees) who may have no prior cybersecurity training, which creates a continuous stream of newly susceptible employees. Hospital systems are vulnerable due to significant end point complexity, a term used to describe the large number of IT devices that could be targeted in an attack. For example, every employee smartphone that is connected to the network is a potential risk, as are other networked devices (eg, patient monitors, clinical workstations, tablets, and all of the core information systems already in use).^21^ In addition, hospital information systems are highly interdependent. An electronic health record is dependent on a laboratory information system to display clinical results. The laboratory information system, in turn, is dependent on a network connection to the laboratory analyzer system to process results. Attacking 1 system could significantly influence multiple downstream systems. Finally, locking down information systems is difficult. In a large health care system, there are typically a vast, heterogeneous, and distributed set of users that need access (eg, affiliated practices, state-level information exchanges, and reporting agencies). It only takes 1 successful phishing email, sent to 1 user, to shut down a critical system, potentially disrupting care across an entire organization.

There are many strategies for preventing or minimizing the consequences of phishing attacks. One strategy is to prevent phishing emails from being received or read in the first place (eg, using technology to filter emails based on patterns suspicious for phishing or modifying emails to indicate they are from external senders). A second strategy is to minimize the value of username and passwords, by requiring multifactor authentication (eg, a unique code generated by a smartphone application that must be entered to log in) or requiring special access controls for specific systems, so that credentials are less useful even if they are obtained. A third strategy is to foster employee awareness and training, and our results suggest that including phishing simulation campaigns as part of employee awareness or training may be helpful. There were several institution-level awareness efforts implemented in conjunction with phishing simulation campaigns. Some examples include distribution of antiphishing laptop decals and multilingual antiphishing posters, as well as phishing awareness in annual employee training programs. These are just some of the components of an information security program, and a robust plan needs to include multiple approaches.

### Limitations

There are several limitations to our study. First, we used a convenience sample of institutions, all of which have an information security organization mature enough to conduct phishing simulations. While not representative of the entire US health care system, we have no reason to believe that the trends described herein would be different at other institutions. Furthermore, the click rate estimates may be conservative because systems with robust information security programs would likely have lower click rates than other institutions. Second, we did not have access to employee-level data (eg, to look at trends based on department, individual employees, or employee characteristics like age, sex, or role in the organization or to look at correlations between individuals because not all employees received all phishing simulation emails). Third, we did not adjust for additional factors that could influence click rates, such as campaign complexity, timing, and other institutional factors like intercampaign training programs or informal awareness efforts. Fourth, we are also unsure of the sustainability of click rate improvements over time.

## Conclusions

In summary, current click rates in phishing simulations at US health care organizations indicate a major cybersecurity risk. These click rates highlight the importance of phishing emails as an attack vector, as well as the challenge of securing information systems. Repeated campaigns were associated with improved click rates, suggesting that simulated phishing campaigns are an important component of a proactive approach to reducing risk. It is necessary for all members of the health care community to understand this risk, particularly as safe and effective health care delivery becomes increasingly dependent on information systems.

## Notes

Supplement. **eTable 1.** Examples of Phishing Emails (Identifying Information Replaced by [Institution]) **eTable 2.** Hospital Characteristics **eTable 3.** US Census Regions and Divisions

## References

[1] Clarke R, Youngstein T Cyberattack on Britain’s National Health Service: a wake-up call for modern medicine. N Engl J Med. 2017;377(5):-. doi:10.1056/NEJMp1706754 28591519

[2] Nigrin DJ When “hacktivists” target your hospital. N Engl J Med. 2014;371(5):393-395. doi:10.1056/NEJMp1407326 25075830

[3] Bai G, Jiang JX, Flasher R. Hospital risk of data breaches. JAMA Intern Med. 2017;177(6):878-880. doi:10.1001/jamainternmed.2017.0336 28384777 PMC5818824

[4] Ponemon Institute LLC Sixth annual benchmark study on privacy & security of healthcare data. http://www.ponemon.org/blog/sixth-annual-benchmark-study-on-privacy-security-of-healthcare-data-1. Published 2016. Accessed October 26, 2018.

[5] Ponemon Institute LLC The cost of phishing & value of employee training. https://info.wombatsecurity.com/hubfs/Ponemon_Institute_Cost_of_Phishing.pdf. Published 2015. Accessed October 26, 2018.

[6] Duncan I, McDaniels AK MedStar hack shows risks that come with electronic health records. *Baltimore Sun* April 2, 2016 http://www.baltimoresun.com/health/bs-md-medstar-healthcare-hack-20160402-story.html. Accessed July 26, 2018.

[7] Gordon WJ, Fairhall A, Landman A Threats to information security: public health implications. N Engl J Med. 2017;377(8):707-709. doi:10.1056/NEJMp1707212 28700269

[8] National Institute of Standards and Technology NISTIR 7298 Rev. 2: glossary of key information security terms. https://csrc.nist.gov/publications/detail/nistir/7298/rev-2/final. Published May 2013. Accessed January 26, 2019.

[9] Wright A, Aaron S, Bates DW The big phish: cyberattacks against U.S. healthcare systems. J Gen Intern Med. 2016;31(10):1115-1118. doi:10.1007/s11606-016-3741-z 27177913 PMC5023604

[10] American Medical Association and Accenture. Taking the physician’s pulse on cybersecurity. https://www.slideshare.net/accenture/taking-the-physicians-pulse-on-cybersecurity. Published 2017. Accessed July 26, 2018.

[11] Hanley JA, Negassa A, Edwardes MD, Forrester JE Statistical analysis of correlated data using generalized estimating equations: an orientation. Am J Epidemiol. 2003;157(4):364-375. doi:10.1093/aje/kwf215 12578807

[12] Path to cyber resilience: sense, resist, react: EY’s 19th Global Information Security Survey 2016-17. https://www.ey.com/Publication/vwLUAssets/Global_Information_Security_Survey_2016/$FILE/REPORT%20-%20EY's%2019th%20Global%20Information%20Security%20Survey.pdf. Accessed January 26, 2019.

[13] PhishMe. Enterprise phishing susceptibility report. https://phishme.com/wp-content/uploads/2017/10/PhishMe_EnterprisePhishingSusceptibilityReport_2015_Final.pdf. Published 2015. Accessed October 26, 2018.

[14] Wirth A Hardly ever a dull moment: the ongoing cyberthreats of 2017. Biomed Instrum Technol. 2017;51(5):431-433. doi:10.2345/0899-8205-51.5.431 28934579

[15] Humer C, Finkle J Your medical record is worth more to hackers than your credit card. https://www.reuters.com/article/us-cybersecurity-hospitals-idUSKCN0HJ21I20140924. Published September 24, 2014. Accessed July 28, 2018.

[16] Stack B Here’s how much your personal information is selling for on the dark web. https://www.experian.com/blogs/ask-experian/heres-how-much-your-personal-information-is-selling-for-on-the-dark-web/. Published April 9, 2018. Accessed July 26, 2018.

[17] Le Bris A, El Asri W. State of cybersecurity & cyber threats in healthcare organizations: applied cybersecurity strategy for managers. https://blogs.harvard.edu/cybersecurity/files/2017/01/risks-and-threats-healthcare-strategic-report.pdf. Published 2017. Accessed July 26, 2018.

[18] KPMG Health care and cyber security: increasing threats require increased capabilities. https://assets.kpmg/content/dam/kpmg/pdf/2015/09/cyber-health-care-survey-kpmg-2015.pdf. Accessed January 26, 2019.

[19] Brudner E Email open rates by industry: see how you stack up. https://blog.hubspot.com/sales/average-email-open-rate-benchmark. Published October 2018. Accessed January 3, 2019.

[20] Collins SK, McKinnies RC, Matthews EP, Collins KS A ministudy of employee turnover in US hospitals. Health Care Manag (Frederick). 2015;34(1):23-27. doi:10.1097/HCM.0000000000000038 25627851

[21] Jalali MS, Kaiser JP Cybersecurity in hospitals: a systematic, organizational perspective. J Med Internet Res. 2018;20(5):e10059. doi:10.2196/10059 29807882 PMC5996174

