# Supplementary material for: Assessment of Employee Susceptibility to Phishing Attacks at US Health Care Institutions
Source: JAMA Netw Open. 2019 Mar 8;2(3):e190393. doi: 10.1001/jamanetworkopen.2019.0393 (PMC6484661; doi:10.1001/jamanetworkopen.2019.0393)
Supplement: Supplement. — eTable 1. Examples of Phishing Emails (Identifying Information Replaced by [Institution]) eTable 2. Hospital Characteristics eTable 3. US Census Regions and Divisions [file jamanetwopen-2-e190393-s001.pdf]

Supplementary Online Content

Gordon WJ, Wright A, Aiyagari R, et al. Assessment of employee susceptibility to phishing attacks at US health care institutions. *JAMA Netw Open*. 2019;2(3):e190393. doi:10.1001/jamanetworkopen.2019.0393

eTable 1. Examples of Phishing Emails (Identifying Information Replaced by [Institution])

eTable 2. Hospital Characteristics

eTable 3. US Census Regions and Divisions

This supplementary material has been provided by the authors to give readers additional information about their work.

© 2019 Gordon WJ et al. *JAMA Network Open*.

eTable 1. Examples of Phishing Emails (Identifying Information Replaced by [Institution]). The first example is a simulated phishing email sent as part of a campaign by one of the collaborating sites. The second example is an actual phishing email received by one of our collaborating sites.

**Email Type**	**Phishing Email Text**
Phishing Simulation	From: [Institution] Support <[institution]-webmail@gmail.com><br><br>Subject: Email Quota Exceeded<br><br>Attention [Institution] User:<br><br>Your [Institution].edu inbox has exceeded the email storage limit currently permitted under Hospital guidelines. You are running out of storage and may not be able to send or receive email your mailbox has been upgraded.<br><br>Please click [here] to upgrade your mailbox.<br><br>Thanks,<br><br>[Institution] Assistant System Administrator
Real Phishing Email	From: support.team@[institution].org<br><br>Subject: Unauthorized Access<br><br>For your [institution].org mailbox account security we recommend that you CLICK HERE and verify your [institution].org account and always exit your [institution].org account using the logout button in the upper right corner instead of just closing the tab of your browser.<br><br>This serves as an additional security measure to prevent unauthorized access to your account.<br><br>[Institution].org Support HelpDesk ©2017. All rights reserved.

© 2019 Gordon WJ et al. *JAMA Network Open*.

eTable 2. Hospital Characteristics**.** Data limited to preserve the identity of individual institutions.

Institution	Geographic Reach	For Profit / Non Profit Status	Annual Revenue
A	Multi-state	Non-Profit	> $10 billion
B	Multi-state	Non-Profit	> $10 billion
C	Single state	Non-Profit	$1-10 billion
D	Multi-state	Non-Profit	$1-10 billion
E	Single state	Non-Profit	$1-10 billion
F	Multi state	Non-Profit	$1-10 billion

© 2019 Gordon WJ et al. *JAMA Network Open*.

eTable 3. US Census Regions and Divisions. Listed are the US census regions represented by the institutions included in this study. Some institutions span more than one census region, therefore the totals are greater than 6.

US Census Region	Count
Northeast	2
South	2
Midwest	2
West	2

© 2019 Gordon WJ et al. *JAMA Network Open*.
